# Mentorship of young researchers in resource-limited settings: experiences of the mentees from selected health sciences Universities in Tanzania

**DOI:** 10.1186/s12909-023-04369-z

**Published:** 2023-05-24

**Authors:** Alex Mremi, Godwin Pancras, Dorah Mrema, Baraka Morris, Tosi Mwakyandile, Delfina R Msanga, James S Mundamshimu, Bartholomeo Nicholaus, Honest H Massawe, Mwita Matiko, Maryam Amour, Evangelista Malindisa

**Affiliations:** 1 Faculty of Medicine, Kilimanjaro Christian Medical University College, P.O. Box 2240, Moshi, Tanzania; 2 School of Public Health and Social Sciences, the Muhimbili University of Health and Allied Sciences, P.O. Box 65001, Dar-es-salaam, Tanzania; 3 Faculty of Nursing, Kilimanjaro Christian Medical University College, P.O. Box 2240, Moshi, Tanzania; 4 School of Nursing, Muhimbili University of Health and Allied Sciences, P.O. Box 65001, Dar es salaam, Tanzania; 5 School of Biomedical Sciences, Campus College of Medicine, Muhimbili University of Health and Allied Sciences, P.O. Box 65001, Dar es Salaam, Tanzania; 6 School of Medicine, the Catholic University of Health and Allied Sciences, P.O. Box 1464, Mwanza, Tanzania; 7 Archbishop Anthony Mayala School of Nursing, the Catholic University of Health and Allied Sciences, P.O. Box 1464, Mwanza, Tanzania

**Keywords:** Mentorship, Research, Mentees, Lesson learned, Tanzania

## Abstract

**Introduction:**

Mentorship is an essential component of research capacity building for young researchers in the health sciences. The mentorship environment in resource-limited settings is gradually improving. This article describes mentees’ experiences in a mentorship program for junior academicians amid the COVID-19 pandemic in Tanzania.

**Methods:**

This is a survey study that examined the experiences of mentees who participated in a mentorship program developed as part of the Transforming Health Education in Tanzania (THET) project. The THET project was funded by the US National Institutes of Health (NIH) under a consortium of three partnering academic institutions in Tanzania and two collaborating US-based institutions. Senior faculty members of respective academic institutions were designated as mentors of junior faculty. Quarterly reports submitted by mentees for the first four years of the mentorship program from 2018 to 2022 were used as data sources.

**Results:**

The mentorship program included a total of 12 mentees equally selected from each of the three health training institutions in Tanzania. The majority (7/12) of the mentees in the program were males. All mentees had a master’s degree, and the majorities (8/12) were members of Schools/Faculties of Medicine. Most mentors (9/10) were from Tanzania’s three partnering health training institutions. All mentors had an academic rank of senior lecturer or professor. Despite the onset of the COVID-19 pandemic, the regular weekly meetings between mentors and mentees were not affected. By the fourth year of the mentorship program, more than three-quarters of mentees had published research related to the mentorship program in a peer-reviewed journal, over half had enrolled in Ph.D. studies, and half had applied for and won competitive grant awards. Almost all mentees reported being satisfied with the mentorship program and their achievements.

**Conclusion:**

The mentorship program enhanced the skills and experiences of the mentees as evidenced by the quality of their research outputs and their dissemination of research findings. The mentorship program encouraged mentees to further their education and enhanced other skills such as grant writing. These results support the initiation of similar mentorship programs in other institutions to expand their capacity in biomedical, social, and clinical research, especially in resource-limited settings, such as Sub-Saharan Africa.

**Supplementary Information:**

The online version contains supplementary material available at 10.1186/s12909-023-04369-z.

## Introduction

The role of mentorship in ensuring that the next generation of researchers is reasonably prepared to handle current, future, and re-emerging global health challenges is very important as research expertise is often the result of good mentorship. Various definitions of mentorship exist. However, in this study, we utilize the one proposed by the Standing Committee on Postgraduate Medical and Dental Education (SCOMPE), which is “A process whereby an experienced, highly regarded, empathetic person (the mentor) guides another (usually younger) individual (the mentee) in the development and re-examination of their ideas, learning, and personal and professional development” [1]. The mentor is often an expert in their field and genuinely invests and becomes interested in their mentee’s goals. A good mentor often encourages open communication, provides structured learning opportunities, and facilitates the career and personal development of the mentee [2, 3]. Therefore, a successful mentor-mentee relationship requires the active participation of both parties, who treat each other as partners in promoting the mentees’ professional development [4, 5].

Within academic institutions, mentorship environments equip junior faculty with the confidence, skills, and knowledge needed to excel in their careers. Thus, effective mentorship is a critical determinant of academic success [6, 7]. Mentorship provides a platform for structured professional growth, as young or emerging researchers in health science institutions navigate between their demanding clinical duties and the demands of establishing their research expertise [7–10]. A well-organized mentor-mentee relationship enables the mentee to easily access and establish networking opportunities and evolve into an independent professional. Additionally, a good mentorship initiative can accelerate research productivity, career development, and academic promotion [11–15].

To date, there is an increasing body of evidence on the success of research mentorship programs [16, 17]. However, most accounts are from the mentors’ points of view [18, 19], and to the best of our knowledge, few mentees’ experiences have been documented [20, 21]. Further, few have described mentorship experiences during the COVID-19 pandemic in resource-limited settings. To address these shortcomings in the research literature, we aimed to describe the experiences of the mentees who participated in the Community of Young Research Peers (CYRP) during the first three and a quarter years of the Transforming Health Education in Tanzania (THET) project [16]. We examined the successes and challenges of the mentorship program and the resilience of the mentees during the COVID-19 outbreak.

## Materials and methods

### Settings and establishment of the mentorship program

This was survey research done to understand CYRP (mentees’) experiences during the implementation of the THET project [22]. THET is a consortium of three health universities in Tanzania, the Muhimbili University of Health and Allied Sciences (MUHAS), Kilimanjaro Christian Medical University College (KCMUCo), and the Catholic University of Health and Allied Sciences (CUHAS), and two partnering US-based institutions, the University of California, San Francisco (UCSF) and Duke University, North Carolina [22]. The overarching aim of the THET project was to use innovative educational strategies to transform health education and produce competent health professionals. One of the project’s sub-aims was geared toward the mentorship of junior faculty members who worked in local academic institutions. The THET project sought to promote research mentorship at higher learning institutions in Tanzania by designing and implementing a mentorship program, Community of Young Research Peers (CYRP). This mentorship program has five foundational principles: (1) to be self-governed by the mentees; (2) to provide peer-to-peer mentoring; (3) to provide mentees with the opportunity to provide mentorship to undergraduate students; (4) to provide mentees with the opportunity to undertake research training; and (5) to provide mentees with the opportunity to participate in mentored research projects [22].

The overarching goals of the CYRP were to: (1) promote peer-to-peer mentoring; (2) promote research training within and outside of the three institutions; (3) elicit innovation and academic outputs; (4) promote new approaches in research and redefine priority research needs, and (5) strengthen multidisciplinary research collaborations, especially in the area of HIV/AIDS. The CYRP mentorship program was divided into two cohorts. The first cohort began in the year 2018 and the second cohort was established in 2022; each cohort had twelve members equally distributed among the three universities. The present study examines only the experiences of the first CYRP cohort. Members of the first CYRP cohort were selected by a committee of senior faculty from the partnering institutions based on strength of the research concept notes submitted by junior faculty members.

Detailed information about CYRP selection and project implementation has been documented previously [22]. All mentees were below 40 years of age at the time of recruitment into the program. The characteristics of the CYRP are shown in Table 1.

**Table 1:** Characteristics of the Mentees’ Cohort

ID	Institution	Sex	Faculty/School	Department	Academic rank
1	KCMUCo	M	Medicine	Pathology	Lecturer
2	MUHAS	M	Public Health and Social Sciences	Bioethics	Assistant lecturer
3	KCMUCo	F	Nursing	Nursing	Assistant Lecturer
4	MUHAS	M	Nursing	Nursing	Assistant Lecturer
5	MUHAS	F	Biomedical sciences	Clinical Pharmacology	Assistant Lecturer
6	CUHAS	F	Medicine	Pediatrics and child health	Lecturer
7	CUHAS	F	Medicine	Physiology	Assistant Lecturer
8	CUHAS	M	Nursing	Clinical Nursing	Assistant Lecturer
9	KCMUCo	M	Medicine	Urology	Lecturer
10	KCMUCo	M	Medicine	Orthopedics	Lecturer
11	CUHAS	M	Medicine	Psychiatry	Lecturer
12	MUHAS	F	Public Health and Social Sciences	Community Health	Assistant Lecturer

### Mentors of the program

A group of senior faculty members was selected from the partnering institutions as mentors based on the following criteria: academic position of senior lecturer or above; expertise in HIV/AIDS research, basic sciences, implementation science, or socio-behavioral sciences; expertise in research methodology and/or data analysis; and willingness to mentor junior faculty members. Ten senior faculty members (three from each of the partner institutions in Tanzania and one from Duke University) constituted the team of mentors for the CYRP (see Table 2). Mentors could mentor mentees in their respective institutions or other partner institutions and were not restricted to a single mentee. Both mentors and mentees were able to choose each other based on their fields of expertise, research interests, and goals.

**Table 2:** Characteristics of the mentors’ cohort included in the mentoring program

ID	Institution	Sex	Faculty/School	Department	Academic rank
1	KCMUCo	F	Medicine	Pediatrics	Associate Professor
2	KCMUCo	M	Public Health	Public Health	Associate Professor
3	KCMUCo	M	Medicine	Urology	Professor
4	MUHAS	M	Biomedical sciences	Physiology	Associate Professor
5	MUHAS	M	Public Health	Community Health	Associate Professor
6	MUHAS	M	Diagnostic medicine	Microbiology and Immunology	Professor
7	CUHAS	M	Medicine	Biochemistry	Senior Lecturer
8	CUHAS	M	Medicine	Microbiology	Professor
9	CUHAS	M	Medicine	Internal medicine	Senior Lecturer
10	Duke	M	Medicine	Medicine	Professor

Mentors were involved in each stage of the mentees’ research projects, from conceptualization, proposal writing, data collection, analysis, review of manuscript drafts, selection of journals, and eventually manuscript submission. Mentors also ensured that studies were done professionally, ethically, and timely, and were scientifically sound. Furthermore, mentors organized and conducted workshops and training to foster the research skills of the CYRP mentees. Some of the areas covered included qualitative and quantitative research methods, secondary data analysis, grant writing, manuscript writing, and scientific presentations.

### Data collection and analysis

For this study, the data was obtained from quarterly progress reports that were submitted by the mentees to the program coordinator. The quarterly reports contained information about the mentees’ research progress, physical meetings with respective mentors, short courses taken, conferences attended, mentorship from non-CYRP faculty members, and mentorship of undergraduate students, as well as challenges faced. We also analyzed the data from anonymous feedback reports of the mentees’ satisfaction with the program, which were obtained from surveys that had been developed by the program coordinators. Data were analyzed using STATA version 17 (StataCorp LLC). Descriptive statistics have been categorized and presented in Tables 1, 2, 3 and 4.

**Table 3:** Progress of the mentees and achievements by the 1st quarter of the 4th year of the mentorship program

ID	All published papers since enrolment into the CYRP*	Years since Ph.D. registration	Successful grant applications	Grants applied and waiting for a response/ not won	Scientific conference presentations	Undergraduate students mentored
1	20 (1)	3	4	3	6	7
2	3 (2)	2	3	1	4	4
3	4 (1)	N/A	1	0	2	4
4	2 (1)	2	0	0	0	5
5	4 (1)	3	1	3	2	0
6	4 (1)	1	1	0	1	5
7	6 (0)	3	1	3	6	7
8	1 (0)	1	0	0	4	1
9	6 (1)	2	0	0	7	7
10	5 (0)	3	0	0	2	3
11	8 (2)	2	0	4	4	4
12	7 (1)	N/A	5	1	5	10

* The Total number of articles published by CYRP as lead or co-author during the mentorship program. Numbers in brackets are the published articles from the mentees’ research that were sponsored by the THET project

**Table 4:** Mentees’ Satisfaction with the mentorship program

Question	Agree	Neutral	Disagree
Are you satisfied with the quality of physical meeting(s) every year?	10/12	2/12	0
Are you satisfied with the quality of the mentor-mentee relationship with your institutional senior leader?	8/12	2/12	2/12
Are you satisfied with the quality of your meeting(s) with the institutional Senior Leader?	9/12	2/12	1/12
Are you satisfied with the quality of your meeting(s) with the primary research mentor?	10/12	2/12	0
Are you satisfied with your institutional administrative staff in assisting with your queries?	8/12	4/12	0
The overall program had a positive effect on your career.	9/12	3/12	0
Are you satisfied with the quality of video conference sessions?	(10/12)	(1/12)	(1/12)
It was easy to find enough time with the primary mentor?	10/12	2/12	0
Did COVID-19 pandemic affect physical meetings with mentors?	12/12	0	0
Are you satisfied with the training program during the mentorship?	7/12	4/12	1/12
Overall, the program goals set were achieved	10/12	2/12	0

## Results

### Mentees’ profiles

We reported the findings from records of the mentees tracked over four years. A total of 12 mentees were enrolled in the first cohort, four from each Tanzanian partnering institution. All mentees were faculty members in their respective institutions. The majority, 7/12 were males, and 6/12 were faculty members in departments in schools of medicine (Table 1).

### Mentors’ profiles

Most of the mentors were male (9/10); 6 of 10 were faculty members in schools of medicine; 2 of 10 were Senior Lecturers, 4 of 10 were associate professors, and 4 of 10 were professors (Table 2).

### Achievements of mentees in the first 4 years of the mentorship project

By the end of the first quarter of the fourth year of the program, most of the mentees (9/12) had at least one published or accepted manuscript out of their respective mentored research projects; 10 of 12 had registered for a Ph.D. fellowship; and 7 of 12 had applied for and received research grants for their research program (Table 3).

### Assessment of the mentees’ satisfaction with the quality of the mentorship program

In Table 4, most mentees (10/12) were satisfied with the quality of physical meetings conducted in the mentorship program; 8 of 12 were satisfied with the quality of the mentor-mentee relationship; 9 of 12 were satisfied with the quality of their meetings with institutional leaders. Most mentees (8/12) were satisfied with the assistance provided by institutional administrative staff regarding their queries, and 9 of 12 felt that the overall program had a positive effect on their careers. Most mentees (10/12) were satisfied with the quality of video conference sessions, and 10 of 12 agreed that it was easy to find enough time with their primary mentors. Seven of 12 mentees were satisfied with the mentorship training program, and most mentees (10/12) noted that overall, the program goals were achieved. Less than half of the mentees (5/12) reported a weekly mentor-mentee interaction, (3/12) had bi-weekly interactions, and (2/12) reported a monthly interaction with their mentor.

### Impact of COVID-19 on the mentorship program

A challenge encountered during the implementation of the mentorship program was the emergency of COVID-19. Globally, during the pandemic, many governments imposed travel restrictions and lockdowns that interrupted physical attendance at international scientific conferences. In Tanzania, the first COVID-19 patient was reported in March 2020 reading to a series of other cases [23]. It was during this time that the government halted all research activities involving physical contact for several weeks. Due to that, the physical meetings between mentors and mentees were halted. Apart from physical meetings, the mentees also suspended some research activities, especially the enrollment of participants and the order of laboratory reagents. To mitigate some of the challenges, mentees willingly switched to virtual meetings with mentors, and the discussions on the individual project progress as well as lectures on topics such as data analysis, scientific paper writing, and presentations were ongoing.

## Discussion

Nurturing the research careers of young investigators is of paramount importance for sustainable growth and ensuring research expertise in resource-limited settings in sub-Saharan Africa. Mentoring is an important component of this endeavor. Mentorship from more experienced academicians and investigators has positive and productive results. The mentorship model in the CYRP program has resulted in mentee success as demonstrated by high outputs in terms of deliverables, which included mentees registering for Ph.D. studies, presenting study findings at national and international scientific conferences, and publishing articles from mentored research projects in international peer-reviewed scientific journals.

Furthermore, the mentees were successful in obtaining external research fellowships and research grants, which sets the ground for their successful future research careers [22]. Additionally, the disposition towards mentorship was established among the mentees, who mentored junior faculty members as well as undergraduate students. These actions support the sustainability of mentorship practices in the three partnering institutions and other similar institutions where the mentees are likely to serve during their careers.

A satisfactory mentor-mentee relationship is dependent on the characteristics of both parties. And according to Straus and colleagues, reciprocity between parties, having shared values and clearly defined goals, and personal connections are critical to a fruitful and satisfactory mentor-mentee relationship [24]. This is consistent with the findings of this study as most mentees reported having a satisfactory relationship with their mentors. Furthermore, a fruitful mentoring relationship ought to be mutually beneficial despite the inherent differences in power and experience that exist between a mentor and a mentee [25]. However, some mentees indicated that they were dissatisfied with the mentorship program in various aspects, including the quality of mentor-mentee relationships with their institutional mentors, the quality of meetings with institutional mentors, video conference sessions, and the overall training during the mentorship program. Similar observations have been reported elsewhere [2, 26–29]. Although these concerns were observed among a few mentees, further research is needed to explore and adequately address the challenges to ensure the future success of similar programs.

Several practical steps can be implemented in similar mentorship programs to address the challenges. For instance, the new mentees can be informed about previous experiences, including the success and challenges, and they can suggest and work on ways to overcome the challenges as well as adopt and improve the previous success strategies. Mentors too, need to be aware of the previous mentees’ and mentors’ experiences and choose the best ways to improve the mentorship program and attain the best out of it. For instance, *Cabana et al.* highlighted that The American Academy of Pediatrics continues to promote and encourage efforts to facilitate the creation of new knowledge and ways to reduce barriers experienced by trainees, practitioners, and academic faculty pursuing research [25]. Continued progress in mentoring new researchers can be made by systematic research revealing best practices for fostering positive relationships. Studies focusing on the most salient challenges of mentees and identifying their effective strategies for working with mentors hold promise for enhancing the education and training of medical researchers.

Moreover, as the impact of COVID-19 was being felt by all works of life, research was not spared. Mentees’ research activities including laboratory, field visits, and face-to-face interviews stalled for a few weeks leading to missed deadlines to accomplish the studies. These shortfalls are also echoed by academicians and researchers in developed countries [30, 31]. And according to Bansal *et al*, the disruption of planned research activities by the COVID-19 outbreak could have equally affected the “lifelong career trajectory” of upcoming researchers [32]. However, more research is still needed to deeply understand its impacts in developing countries and how it has reshaped our approach to health research.

This study possesses some methodological limitations. It used data collected from progress reports submitted quarterly by the mentees (CYRPs) as part of the THET project management. This might have contributed to missed information and a narrow focus on understanding mentees’ experiences and satisfaction with the mentorship program. However, this is a limitation of many studies utilizing secondary data. Secondly, the authors of this study were also the mentees from whom the reports were collected during the mentorship period which could lead to a potential bias. However, this was mitigated by ensuring that only data contained in the progress reports were reported.

### Conclusion

Mentoring early career investigators through the multidisciplinary CYRP was a viable model for the growth and development of research expertise. The mentorship program enriched the skills and experiences of the mentees and enhanced the quality of their research outputs, resulting in the dissemination of research findings at international conferences and peer-reviewed publications. We recommend similar mentorship programs to other institutions of higher learning to increase the number of young faculty from diverse backgrounds in health sciences. This could sustainably expand the capacity of clinical research for the present and future generations, especially in Sub-Saharan Africa where resources are limited.

## Electronic supplementary material

Below is the link to the electronic supplementary material.

Supplementary Material 1

## Data Availability

Data are available from the corresponding author upon reasonable request.

## List of Abbreviations

CUHAS: Catholic University of Health and Allied Sciences.
KCMUCo: Kilimanjaro Christian Medical University College.
MUHAS: Muhimbili University of Health and Allied Sciences.
SCOMPE: Standing Committee on Postgraduate Medical and Dental Education.
CYRP: Community of Young Research Peers.
THET: Transforming Health Professions Education in Tanzania.
UCSF: University of California, San Francisco.

## References

[1] SCOPME. 1998: SCOPME (Standing Committee on Postgraduate Medical and Dental Education). Supporting Doctors and Dentists at Work: An Enquiry into Mentoring. SCOPME, London 1998)

[2] Straus SE, Chatur F, Taylor M (2009). Issues in the Mentor-Mentee Relationship in academic medicine: a qualitative study. Acad Med.

[3] Katzka DA (2017). How to balance clinical and research works in the current era of academic medicine. Gastroenterology.

[4] DuBois DL, Holloway BE, Valentine JC, Cooper H (2002). Effectiveness of mentoring programs for youth: a meta-analytic review. Am J Community Psychol.

[5] Bixen CE, Papp KK, Hull AL (2007). Developing a mentorship program for clinical researchers. J Contin Educ Health Prof.

[6] Rosenkranz SK, Wang S, Hu W (2015). Motivating medical students to do research: a mixed methods study using self-determination theory. BMC Med Educ.

[7] Steiner J, Curtis P, Lanphear B, Vu K, Main D (2004). Assessing the role of influential mentors in the research development of primary care fellows. Acad Med.

[8] Winter FD (2007). Einstein: his life and Universe by Walter Isaacson. Proc (Baylor Univ Med Cent).

[9] Karcher MJ, DuBois DL, Karcher MJ (2005). Cross-age peer mentoring. Handbook of youth mentoring.

[10] Karcher MJ (2007). Research in action: cross-age peer mentoring.

[11] Sambunjak D, Strauss SE, Marusic A (2006). Mentoring in academic medicine a systematic review. JAMA.

[12] Johnson MO, Subak LL, Brown JS (2010). An innovative program to train health sciences researchers to be effective clinical and translational research mentors. Acad Med.

[13] Sakushima K, Mishina H, Fukuhara S (2015). Mentoring the next generation of physician-scientists in Japan: a cross-sectional survey of mentees in six academic medical centers. BMC Med Educ.

[14] Taylor J (2003). Training new mentees: a manual for preparing youth in mentoring programs.

[15] Ley TJ, Rosenberg LE (2005). The physician-scientist career pipeline in 2005: build it and they will come. JAMA.

[16] Balandya E, Sunguya B, Kidenya B, Nyamhanga T, Minja IK, Mahande M, Mmbaga BT, Mshana SE, Mteta K, Bartlett J, Lyamuya E. Joint Research Mentoring through the community of Young Research Peers: a case for a Unifying Model for Research Mentorship at higher Learning Institutions. Adv Med Educ Pract 2022 Apr 21;13:355–67. DOI: 10.2147/AMEP.S356678. PMID: 35478975; PMCID: PMC9038151. 10.2147/AMEP.S356678 PMC9038151 35478975

[17] Taylor CA, Taylor JC, Stoller JK. The influence of mentorship and role modeling on developing Physician–Leaders: views of aspiring and established physician–leaders. J Gen Intern Med. 2009 Oct;24(10):1130–4. 10.1007/s11606-009-1091-9 PMC2762511 19711134

[18] Carey EC, Weissman DE. Understanding and finding mentorship: a review for Junior Faculty. J Palliat Med. 2010 Nov;13(11):1373–9. 10.1089/jpm.2010.0091 PMC3000901 21091022

[19] Keller TE, Collier PJ, Blakeslee JE, Logan K, McCracken K, Morris C (2014). Early career mentoring for translational researchers: mentee perspectives on challenges and issues. Teach Learn Med.

[20] Chien E, Phiri K, Schooley A, Chivwala M, Hamilton J, Hoffman RM. Successes and Challenges of HIV Mentoring in Malawi: The Mentee Perspective. PLoS One. 2016 Jun 28;11(6):e0158258. DOI: 10.1371/journal.pone.0158258. PMID: 27352297; PMCID: PMC4924818. 10.1371/journal.pone.0158258 PMC4924818 27352297

[21] Alidina S, Tibyehabwa L, Alreja SS (2021). A multimodal mentorship intervention to improve surgical quality in Tanzania’s Lake Zone: a convergent, mixed methods assessment. Hum Resour Health.

[22] Balandya E, Sunguya B, Gunda DW, Kidenya B, Nyamhanga T, Minja IK et al. Building sustainable research capacity at higher learning institutions in Tanzania through mentoring of Young Research Peers. BMC Med Educ. 2021 Mar 17;21(1):166. DOI: 10.1186/s12909-021-02611-0. PMID: 33731103; PMCID: PMC7967782. 10.1186/s12909-021-02611-0 PMC7967782 33731103

[23] Buguzi S. Covid-19: Counting the cost of denial in Tanzania. BMJ. 2021 Apr 27;373:n1052. doi: 10.1136/bmj.n1052. PMID: 33906903. 10.1136/bmj.n1052 33906903

[24] Straus SE, Johnson MO, Marquez C, Feldman MD. Characteristics of successful and failed mentoring relationships: a qualitative study across two academic health centers. Acad Med. 2013 Jan;88(1):82–9. 10.1097/ACM.0b013e31827647a0. PMID: 23165266; PMCID: PMC3665769. 10.1097/ACM.0b013e31827647a0 PMC3665769 23165266

[25] Committee on Pediatric Research (2014). Promoting education, mentorship, and support for pediatric research. Pediatrics.

[26] Leary JC, Schainker EG, Leyenaar JK. The unwritten rules of mentorship: facilitators of and barriers to effective mentorship in Pediatric Hospital Medicine. Hosp Pediatr. 2016 Apr;6(4):219–25. 10.1542/hpeds.2015-0108. Epub 2016 Jan 1. PMID: 26939592; PMCID: PMC5578603. 10.1542/hpeds.2015-0108 PMC5578603 26939592

[27] Humphrey HJ (2010). Mentoring in academic medicine.

[28] Muñoz de Nova JL, Ortega-Gómez M, Abad-Santos F. Research during the SARS-CoV-2 pandemic. Med Clin (Barc). 2021 Jan 8;156(1):39–40. English, Spanish. DOI: 10.1016/j.medcli.2020.09.001. Epub 2020 Sep 25. PMID: 33268132; PMCID: PMC7518177. 10.1016/j.medcle.2020.09.001 PMC7713575 33294623

[29] Zerzan JT, Hess R, Schur E, Phillips RS, Rigotti N (2009). Making the most of mentors: a guide for mentees. Acad Med.

[30] Bratan T, Aichinger H, Brkic N, Rueter J, Apfelbacher C, Boyer L, Loss J. Impact of the COVID-19 pandemic on ongoing health research: an ad hoc survey among investigators in Germany. BMJ Open. 2021 Dec 6;11(12):e049086. doi: 10.1136/bmjopen-2021-049086. PMID: 34872995; PMCID: PMC8649878. 10.1136/bmjopen-2021-049086 PMC8649878 34872995

[31] Hogg HDJ, Low L, Self JE, Royal College of Ophthalmologists’ Academic and Research Subcommittee, Rahi JS. Impact of the COVID-19 pandemic on the research activities of UK ophthalmologists. Eye (Lond). 2022 Oct 31:1–6. doi 10.1038/s41433-022-02293-y. Epub ahead of print. PMID: 36316557; PMCID: PMC9628368. 10.1038/s41433-022-02293-y PMC9628368 36316557

[32] Bansal A, Abruzzese GA, Hewawasam E, Hasebe K, Hamada H, Hoodbhoy Z, Diounou H, Ibáñez CA, Miranda RA, Golden TN, Miliku K, Isasi CR. Impact of COVID-19 pandemic on research and careers of early career researchers: a DOHaD perspective. J Dev Orig Health Dis. 2022 Dec;13(6):800–805. doi 10.1017/S2040174422000071. Epub 2022 Mar 4. PMID: 3524121. 10.1017/S2040174422000071 35241213

