# Supplementary material for: Mentorship of young researchers in resource-limited settings: experiences of the mentees from selected health sciences Universities in Tanzania
Source: BMC Med Educ. 2023 May 24;23:375. doi: 10.1186/s12909-023-04369-z (PMC10206589; doi:10.1186/s12909-023-04369-z)
Supplement: Supplementary file 1 — Supplementary Material 1 [file 12909_2023_4369_MOESM1_ESM.docx]

| Supplementary table 1: **Table 3:** **Progress of the mentees and achievements by the end of 3^rd^ year of the program** | | | |
| --- | --- | --- | --- |
| Mentee ID | Published work | Grant won | Scientific conferences attended |
| 1 | DOI: [10.1016/j.ctarc.2020.100213](https://doi.org/10.1016/j.ctarc.2020.100213) | -CIRGO (https://www.cirgo.org/grants/)  -Pfizer (https://www.pcf.org/)  -MITS (<https://mitsalliance.org/>)  -International Cancer Institute (https://intercancer.com/) | -MUHAS scientific conference (2021)  -NCD/MoH conference (2022)  -BSU-III KCMUCo symposium (2022)  - 1^st^ Tanzania International Cancer Conference (2022)  -USCAP (2022)  -AORTIC (2019) |
| 2 | BMC Medical Ethics 1–10. <https://doi.org/10.1186/s12910-021-00737-w>  BMJ Open 2020;10:e035368. doi:10.1136/bmjopen-2019-035368 | - Amne-Salim COVID-19 Grant - MUHAS- Sida Seed Grants - HIV Research Trust Scholarship grant | - 14^th^ -15^th^ July 2022: **The 10th MUHAS Scientific Conference**. Theme: The role of the health system research in promoting and sustaining universal health coverage, leveraging lessons from the COVID-19 pandemic. Dar es Salaam, Tanzania (Physical; oral)- *10 CPD points awarded* - 17th-19th May 2022: NIMR **31st Annual Joint Scientific Conference**. Theme: A Multisectoral Approach for Health: An Agenda for Health Systems Strengthening Towards Achieving Universal Health Coverage. Tanzania (physical; oral) - 24^th^- 26th May 2022: **Regional Conference on Strengthening Research Ethics (STReK)**. Theme: Strengthening Research Ethics Capacity and Networks. Kenya(virtual; oral) - 17th-19th November 2021: **8th East African Health and Scientific Conference**. By East African Health Research Commission (EAHRC). Theme: East African Community Sustainable Development Goal on Health: Reflection and Path Ahead to 2030. Kenya (virtual; poster) |
| 3 | <https://doi.org/10.3389/fonc.2022.957325> |  | MUHAS Scientific Conference 2021, 3^rd^ National NCD conference 2021 |
| 4 | doi:10.1136/bmjopen-2019-035368 |  |  |
| 5 | http://bmjopen.bmj.com/cgi/content/full/bmjopen-2021-049330 | MUHAS- Sida Seed Grants 2021 | -MUHAS Scientific Conference 2021  -Kumamoto AIDS Seminar 2022 |
| 6 | [**https://doi.org/10.3390/pathogens11030379**](https://doi.org/10.3390/pathogens11030379) | EKC  (https://www.wuerzburg-mwanza.de/1-education) | Advances in Neonatal Medicine (2021) |
| 7 |  | TMA2017GSF-1965-REEHAD-PhD studentship 2019 | MUHAS Scientific Conference 2022: CUHAS Scientific Conference 2021: National Institute for Medical Research AJSC 2022: 3^rd^ National NCD conference 2021: 4^th^ National NCD conference 2022: East African Health and Scientific conference 2021 |
| 8 |  |  | MUHAS Scientific Conference 2021, CUHAS Scientific Conference 2021, 3^rd^ National NCD conference 2021and African Federation of Critical care nurses 2022 |
| 9 | doi:  10.2147/IDR.S29  9776 . |  | Association of urology of Tanzania conference 2022,  4th National NCD conference 2022,  First Tanzanian international cancer conference 2022,  PCF Retreat conference 2022,  COSECSA AGM meeting and scientific conference  2021, 2022  Eschleman workshop and conference 2019,  MUHAS scientific conference 2021 |
| 10 |  |  | 31^st^ Annual Joint Scientific Conference of the National Institute for Medical Research (NIMR), Julius Nyerere Convention Center 17^th^ To 19^th^ May 2022.  MUHAS scientific conference 2021 |
| 11 | <https://doi.org/10.11604%2Fpamj.2021.38.258.27023>  https://doi.org/10.1016/j.jadr.2021.100124 |  | MUHAS scientific conference 2021  WASAD conference, Wurzburg German, 2019 |
| 12 | <https://doi.org/10.1371/journal.pone.0268685>  <https://doi.org/10.1002/jia2.25886>  <https://doi.org/10.3390/tropicalmed7040052>  <https://doi.org/10.3389/fonc.2022.957325>  <https://doi.org/10.3390/vaccines11020465>  <https://doi.org/10.1186/s12879-020-05331-w>  <https://doi.org/10.1016/j.vaccine.2020.09.055> | NIH : 1R21AI166523-01A1 : Serial IGRA testing of Tanzanian adolescents to detect TB in household contacts  MUHAS- Sida Seed Grants : Bone mineral density and serum bone mineral biochemical markers among patients with end stage renal disease at Muhimbili National Hospital in Dar es salaam  UNICEF :COVID-19 vaccine acceptability and associated factors among health care workers in Tanzania  UNICEF & Amref : COVID-19 vaccine acceptability and associated factors among community in Tanzania  **NIH Fellow HIV Implementation Science grant : TB disease and TB Preventive Treatment uptake among adolescents living with HIV attending CTC clinics in Dar-es-Salaam, Tanzania** | AIDS Conference 2022. International AIDS Society. Montreal, Canada. Abstract: Adherence to Antiretroviral Therapy by Medication Possession Ratio and Virological Suppression among Adolescents and Young Adults Living with HIV in Dar es Salaam, Tanzania. August 2022   1. Young Physician leader experience :Regional World Health Summit, Rome June 2023 2. The 8^th^ Tanzania Health Summit. Invitro filtration efficiency for selected face masks to bacteria with a size smaller than SARS-CoV-2 respiratory droplet. October 2021 3. The **International Workshop on HIV & Adolescence 2021.** Predictors of mortality among adolescents and young adults living with HIV on antiretroviral therapy in Dar es Salaam, Tanzania. October 2021   The 25th Annual Conference of the Union-North America. DAR-901 vaccine for the prevention of infection with Mycobacterium tuberculosis among BCG-immunized adolescents in Tanzania: A randomized controlled, double-blind phase 2b trial. Feb 2021 |
